# Application of response surface methodology for COD and ammonia removal from municipal wastewater treatment plant using acclimatized mixed culture

**DOI:** 10.1016/j.heliyon.2022.e09685

**Published:** 2022-06-08

**Authors:** Amirah Ya'acob, Norazwina Zainol, Nor Hazwani Aziz

**Affiliations:** a College of Engineering, Universiti Malaysia Pahang, Lebuhraya Tun Razak, 26300 Gambang, Pahang, Malaysia; b Earth Resources and Sustainability Centre (ERAS), Universiti Malaysia Pahang, 26300 Gambang, Kuantan, Pahang, Malaysia

**Keywords:** Mixed culture, Biological treatment, Municipal wastewater, Ammonia-N, COD, Response surface methodology

## Abstract

This study aimed to optimize conditions influencing the removal of chemical oxygen demand (COD) and ammonia-N in municipal wastewater by using acclimatized mixed culture (AMC). Two-level factorial analysis was used to investigate the factors affecting the degradation of COD and ammonia-N (%); ratio of synthetic wastewater (SW) to acclimatized mixed culture (AMC) (1:1 and 3:1), presence and absence of support media (Yes and No), agitation (0 rpm and 100 rpm) and hydraulic retention time (HRT) (2 and 5 days). A central composite design (CCD) under response surface methodology (RSM) determined the optimum agitation (0 rpm and 100 rpm) and retention time (2 and 5 days). The best conditions were at 3:1 of SW: AMC ratio, 100 rpm agitation, without support media, and 5 days retention time. COD and ammonia-N removal achieved until 57.23% and 43.20%, respectively. Optimization study showed the optimum conditions for COD and ammonia-N removal were obtained at 150 rpm agitation speed and 5 days of retention time, at 70.41% and 64.29% respectively. This study discovers the conditions that affect the COD and ammonia-N removal in the municipal wastewater using acclimatized mixed culture.

## Highlights

• Removal of COD and ammonia-N using acclimatized mixed culture.
• Central composite design was used to optimize process condition.
• The maximum COD removal was found to be 70.41%.
• The maximum ammonia-N removal was found to be 64.29%.

## Graphical abstract

**Figure:**
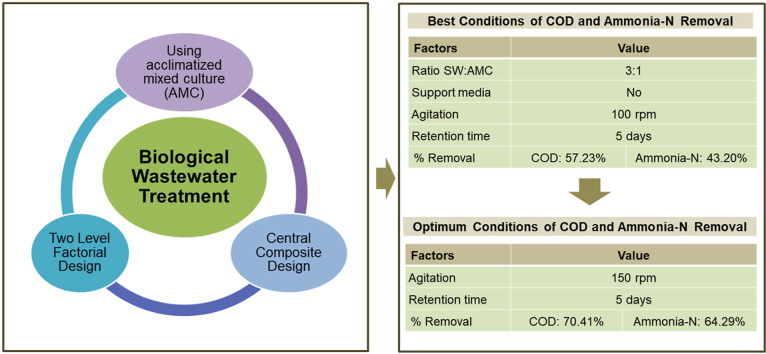

## 1 Introduction

Municipal wastewater is classified by the high content of particulate organic matter and low organic strength (Sikosana et al., 2019). This type of wastewater can provoke human health issues as it can be hazardous when exposed numerously to the surrounding (Khalid et al., 2018). It is then compulsory to reduce the exposure of wastewater for the public's safety and healthiness, and welfare (Khaliq et al., 2017). Generally, the typical effluent's nutrient contained in municipal wastewater produces high concentrations or amounts of COD and ammonia- N. In the municipal industry, the costs of operation are the expenses associated with the monitoring, maintenance, and operation of the plant. Evaluating operating costs during the process alternatives is crucial since the management cost is expensive and poses problems concerning how to finance it (Moral Pajares et al., 2019). Alternatively, the biological treatment, on the other hand, needs only a low quantity of chemicals and energy to reach higher efficiency in removing nutrients and organic carbon. These are, therefore, lead to a better cost-effective and environmental-friendly method for municipal wastewater treatment (Rajasulochana and Preethy, 2019). In biological treatment, a mixed culture is used instead of a single culture to reduce COD and ammonia-N concentration. The use of mixed culture as an alternative method for biological treatment in WWTP has widely been used in the industry nowadays (Samer, 2015). The biological treatment of wastewater has been proven to need a small number of chemicals and energy to reach higher efficiency to remove nutrients and organic carbon. Thus, the apparent economic advantage of biological treatment over other treatment methods such as chemical oxidation is a capital investment and operating costs. Biological treatment is preferable since it relies on microorganisms or mixed culture, to be specific, in breaking down organic wastes using normal cellular processes (Fluence News Team, 2017) to reduce the concentration of COD and ammonia- N.

Studies have demonstrated that the application of mixed cultures over pure cultures in the degradation of pollutants offers advantages, possibly incorporated with the synergistic interactions between the associated members (Constanza et al., 2019). Research also has shown a few typical factors that affect wastewater treatment. Agitation affects the maximum reduction of organic substances in treated sludge. The performance of COD and ammonia-N in municipal wastewater was found to be the highest in 12 days retention time with 95% removal (Sa'at et al., 2019). The total nitrogen removal efficiency was improved by retention time reduction in municipal wastewater, which increases from 80% to 86% (Belli et al., 2017). Additionally, as Salama et al. (2014) mentioned, certain support media provide potential advantages for removing COD and BOD_5_ from municipal wastewater in Morocco, displaying that COD removal goes up to 67.76%, particularly in the biological treatment with PVC media.

One of the most common and powerful experimental designs is full factorial design, in which simulation runs are performed at all combinations of factor levels. The interaction effects of the factors on a response can be investigated by using the full factorial designs. The effect of each factor on the response variable is studied by changing the level of that factor (Jamshidnezhad, 2015). Rahman (2019) mentioned that a full factorial design sometimes seems to be tedious and needs many samples. A fractional design would allow the reduction of experiments from the full factorial with the sacrifice in minor higher-level interaction and nonlinearity effects. Fractional factorial designs are the most widely and commonly used types of design in the industry (Jamshidnezhad, 2015). Analysing one process may be costly, time-consuming, and require much energy as the factors may influence the process. Hence, it is helpful to apply the RSM in designing an experiment, evaluate the factors, and determine optimum conditions for the responses. This study aimed to discover the factors that significantly affect the COD and ammonia-N removal in the municipal wastewater through acclimatized mixed culture and to optimize the condition of biological processes in municipal wastewater using AMC.

## 2 Materials and method

### 2.1 Materials collection

The sampling of both municipal wastewater and mixed culture was conducted from a municipal wastewater pond located at Seremban, Negeri Sembilan. The municipal wastewater was sampled from the pond, while mixed culture was taken from the pond's sediment. The stones ranged from diameter 1.3–2.0 cm each were brought from the store.

### 2.2 Preparation of synthetic wastewater

The synthetic wastewater was prepared based on the contents of COD and ammonia-N in the analyzed sample. Fertilized ammonia-N was added into the municipal wastewater if the COD and ammonia-N content was too low. These are to achieve the COD and ammonia-N design parameters of 449 mg/L and 1000 mg/L, respectively.

### 2.3 Mixed-cultures acclimatization

Acclimatization was carried out to ensure the mixed culture's adaptations to the concentration of total COD and ammonia-N in the 5000 mL acclimatized reactor. The nutrient stock was prepared by using 1.4 g of commercial nutrient stock (CNS) that dissolved in distilled water of 1875 mL and added daily with 1250 mL of mixed culture for the first week in the acclimatized reactor. For the second week, 1.4 g of CNS was mixed with 26 g of fertilized ammonia-N with distilled water to form 1875 mL of synthetic wastewater and was added daily into the acclimatized reactor, with the concentration of COD and TSS were 449 mg/L and 2188 mg/L, respectively. This procedure was carried out daily for two weeks, with 267 mL of CNS prescribed daily for the first week and 267 mL of CNS with fertilized ammonia-N administered daily for the second week.

### 2.4 Preliminary experiment

Synthetic wastewater was newly prepared to substitute the wastewater collected from Cenviro Sdn Bhd for the preliminary experiment. The influence of retention time on COD and ammonia-N removal were investigated in the preliminary experiment. The experiment was performed in accordance with the best condition obtained from a previous study of COD and ammonia-N for municipal wastewater as displayed in Table 1. 187 ml of synthetic wastewater (SW) and 63 ml of acclimatized mixed culture (AMC) were mixed to produce a 250 ml volume mixture according to retention time. The samples were tested using a DR3900 HACH spectrophotometer after five days to obtain the COD and ammonia-N removal value.

**Table 1:** Best condition of COD and ammonia-N for municipal wastewater.

Factors	Range
SW: AMC Ratio	3:1
Support Media	No
Agitation	100 rpm
Retention time	5 days

### 2.5 Experimental setup for factorial analysis

Table 2 shows the selected factors and ranges of municipal wastewater treatment. The factors include SW to AMC ratio (1:1 and 3:1), presence and absence of support media, agitation (0 rpm and 100 rpm), and retention time (2 and 5 days). A new SW mixture was prepared for the factorial analysis experiment using fertilized ammonia-N to replace municipal wastewater from Cenviro Sdn Bhd. The experiments were run in two cycles: 2 days for the first cycle and 5 days for the second cycle. For the first cycle, 8 conical flasks were prepared and divided into 4 by 4 flasks of each ratio. For the 1:1 ratio, the first and third flasks were filled with 85 mL of AMC and 85 mL of SW, while the second flask was filled with 135 mL of AMC and 135 mL of SW. The fourth flask was filled with 125 mL of AMC and 125 mL of SW. For the 3:1 ratio, the first and third flasks were filled with 42.5 mL of AMC and 127.5 mL of SW, while the second flask was filled with 67.5 mL of AMC and 202.5 mL of SW. The 4th flask was filled with 62.5 mL of AMC and 187.5 mL of SW. The initial reading was taken from the solution prepared in the second flask for both ratios. The stone was used as support media for the factorial analysis experiment. 80 mL of stones were added into 4 beakers based on the Design-Expert table. The experiment was conducted in two conditions which were with agitation (100 rpm) and without agitation (0 rpm). 4 conical flasks with agitation were left on an orbital shaker for 2 days with 100 rpm. For the second cycle, the same procedure was performed, and the sample was tested after 5 days of retention time. The experiment was carried out in accordance with the setup generated by Design-Expert software, as shown in Table 3, with all factors being randomized. The data was then analysed to find the most important elements. COD and ammonia-N removal were the two responses.

**Table 2:** Selected factors and their range.

Factors	Low level	High level
Ratio SW:AMC	1:1	3:1
Support media	No	Yes
Agitation (rpm)	0	100
Retention time (days)	2	5

**Table 3:** Experimental setup by two-level factorial design (TLFD).

Std	Factors			
	A	B	C	D
1	1:1	Yes	0	2
2	3:1	Yes	0	2
3	1:1	No	0	2
4	3:1	No	0	2
5	1:1	Yes	100	2
6	3:1	Yes	100	2
7	1:1	No	100	2
8	3:1	No	100	2
9	1:1	Yes	0	5
10	3:1	Yes	0	5
11	1:1	No	0	5
12	3:1	No	0	5
13	1:1	Yes	100	5
14	3:1	Yes	100	5
15	1:1	No	100	5
16	3:1	No	100	5

A: SW: AMC ratio, B: support media, C: Agitation (rpm), D: Retention time (days).

### 2.6 Experimental setup for optimization

The experiments were designed according to CCD with two factors to determine the optimum process parameters. Two factors chosen were; agitation with the range of 50–150 rpm and retention time which ranges from 4 to 6 days. The best condition for COD and ammonia-N removal were utilized to determine the selected value of center point, which is 100 rpm agitation speed and 5 days retention times for CCD experimental setup. The experiments were conducted according to the optimization table (Table 4), with SW: AMC and support media being kept constant during the experiment.

**Table 4:** Experimental setup for optimization experiment.

Std	Run	Factor	
		Agitation (rpm)	Retention Time (days)
1	2	75	4.5
2	4	125	4.5
3	13	75	5.5
4	10	125	5.5
5	9	50	5
6	11	150	5
7	5	100	4
8	12	100	6
9	1	100	5
10	8	100	5
11	6	100	5
12	7	100	5
13	3	100	5

### 2.7 Total solids analysis

A dry weight measurement method was used to determine the total solids analysis (Equation 1). Firstly, the empty aluminium cup was heated up in the oven for 1 hour at 105 °C to make sure there was no moisture content available. The weight of the dried aluminium cup was then recorded. Following that, 50 mL of sample (mixed culture) was placed in an aluminium cup and weighted. The sample was then placed in the oven again for 1 hour at 105 °C until it dry.

(1) $$Total\;Solids=\left(\frac{Weight\;of\;dried\;sample-Weight\;of\;empty\;aluminium\;cup}{Volume\;of\;the\;Sample}\right)$$

### 2.8 Removal analysis of COD

The USEPA Reactor Digestion Method was used to calculate COD removal (Method 8000) (Massalha et al., 2020). The mixture of synthetic wastewater (SW) and acclimatized mixed culture (AMC) was then analyzed using a HACH Spectrophotometer to determine COD removal.

### 2.9 Removal analysis of ammonia-N

The removal of ammonia-N was determined through the Salicylate method (Method 10031). Both acclimatized mixed culture (AMC) and synthetic wastewater (SW) were diluted with deionized water by 100 dilution factors due to the high range of ammonia-N in the mixture. The removal of ammonia-N was then determined by using HACH Spectrophotometer.

### 2.10 Data analysis

The data collected were tabulated in the Design-Expert software. The best condition and optimum conditions for removal of both COD and ammonia-N were analyzed using Design-Expert software.

### 2.11 Validation studies

A validation experiment was performed to validate the result obtained with COD and ammonia-N, both at maximum value. A comparison was made between the experimental and predicted value in order to justify the validity of the model.

## 3 Results and discussion

### 3.1 Preliminary experiments

**Figure 1:**
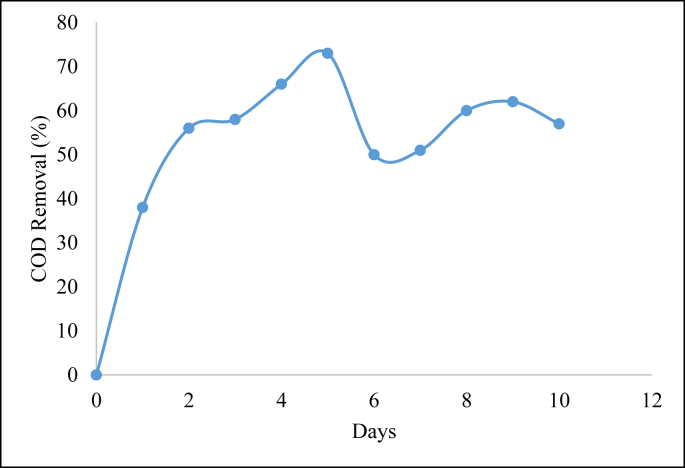
Daily COD removal test reading.

The reading of COD removal for the preliminary study was taken from a sample from day 0 till day 10. Figure 1 exhibited the COD test reading for each day accordingly. The determination of retention time for the factorial analysis was based on the result of the COD removal. The COD removal percentage increased from day 2 until day 5 as shown in Figure 1. These may be due to the consumption of COD by AMC and the suitable reaction between AMC and SW in those ranges. The removal percentage began to fluctuate from day 6 to day 10. The inconsistency of AMC in treating SW in a much longer time might be the reason for the fluctuated removal of COD. The COD removal obtained from this experiment was 73% when using mixed culture. The removal of COD obtained by Neoh et al. (2016) was only 71% when using a pure culture of Ochrobactrum sp. for palm oil mill effluent (POME) under the aerobic condition. The treatment with the mixed culture of Scirpus grossus and *Iris pseudacorus* exhibited a better removal than its single culture where the removal of COD and BOD was up to 89% and 97%, respectively Batik wastewater treatment using the intermittent method (Ladu et al., 2014).

To sum up, day 5 displayed the highest removal percentage of COD in the preliminary experiment. Since all samples took a longer time to remove all COD in wastewater, the experiment was discontinued after 10 days. An increase in COD removal from day 2 to day 5, which was 56% and 73%, respectively, represents the positive removal of COD in the municipal wastewater. Hence, 2 days and 5 days were selected and used for factorial study.

### 3.2 Factorial design screening and analysis

Table 5 presented 16 experimental runs and the removal percentage of COD and ammonia-N. ANOVA was used to analyze the responses based on a 95% confidence level. The removal of COD ranges from 13.89% to 95.12 %, while for ammonia-N, the range is from 12.50% to 66.67%. The lowest percentage of COD removal was recorded in a condition of 1:1 SW: AMC ratio, without support media and agitation with 2 days of retention time. Meanwhile, the highest recorded removal of COD was obtained in an experimented condition of 1:1 SW: AMC ratio, with support media, 0 rpm agitation, during 5 days of retention time. For ammonia-N removal, the lowest percentage value was obtained in a condition of 1:1 SW: AMC ratio, with support media, without agitation and 5 days of retention time. The highest value of ammonia-N removal was obtained in a condition of 3:1 SW: AWC ratio, no support media, 0 rpm agitation, and retention time of 2 days.

**Table 5:** Experimental results of COD and ammonia-N removal (%).

Std	Factors				COD removal (%)	Ammonia-N removal (%)
	A	B	C	D		
1	1:1	Yes	0	2	23.9	14.28
2	3:1	Yes	0	2	35.94	16.67
3	1:1	No	0	2	13.89	42.86
4	3:1	No	0	2	47.4	66.67
5	1:1	Yes	100	2	63.18	42.86
6	3:1	Yes	100	2	53.65	16.67
7	1:1	No	100	2	69.04	28.57
8	3:1	No	100	2	71.72	16.67
9	1:1	Yes	0	5	95.12	12.50
10	3:1	Yes	0	5	62.84	20.00
11	1:1	No	0	5	84.02	25.00
12	3:1	No	0	5	54.12	40.00
13	1:1	Yes	100	5	71.89	12.50
14	3:1	Yes	100	5	61.98	40.00
15	1:1	No	100	5	90.39	12.50
16	3:1	No	100	5	54.28	40.00

A: SW: AMC ratio, B: support media, C: Agitation (rpm), D: retention time (days).

### 3.3 Factor screening for COD removal

The contributions of each factor on COD removal were A – Ratio SW: AMC (4.09%), B – Support Media (0.23%), C – Agitation (11.96%) and D – Retention Time (32.47%). The retention time contributed the most while support media exhibited the least contribution for COD removal. The optimum hydraulic retention time and temperature observed for municipal wastewater in China for the application of an up-flow anaerobic filter (UAF) reactor using mixed culture were 3 days and 33.7 °C with the maximum average COD removal of 91% (Ladu et al., 2014). Devi et al. (2008) discovered a good trend for the percent COD and BOD concentration reduction in municipal wastewater with a higher agitation speed. The COD removal from that research was 92% and, the agitation speed used was 150 rpm Table 6 summarizes the ANOVA for COD removal, which functions to evaluate the model's coefficient, verify the significance of parameters, and designate the strength of interaction for each parameter. The R^2^ for this model is 0.9156, suggesting a good fit model by Olmez (2009) which mentioned that R^2^ should be at least 0.80.

**Table 6:** ANOVA table of COD removal (%).

Source	Sum of squares	Df	Mean squares	F value	P-value
Model	6764.86	9	751.65	7.23	0.0128
A	301.89	1	301.89	2.91	0.1392
B	16.73	1	16.73	0.16	0.7021
C	883.58	1	883.58	8.50	0.0268
D	2399.04	1	2399.04	23.09	0.0030
AC	82.08	1	82.08	0.79	0.4083
AD	1348.73	1	1348.73	12.98	0.0113
BC	176.23	1	176.23	1.70	0.2406
BD	73.96	1	73.96	0.71	0.4312
CD	1482.64	1	1482.64	14.27	0.0092

A: SW: AMC ratio, B: support media, C: Agitation (rpm), D: retention time (days).

**Figure 2:**
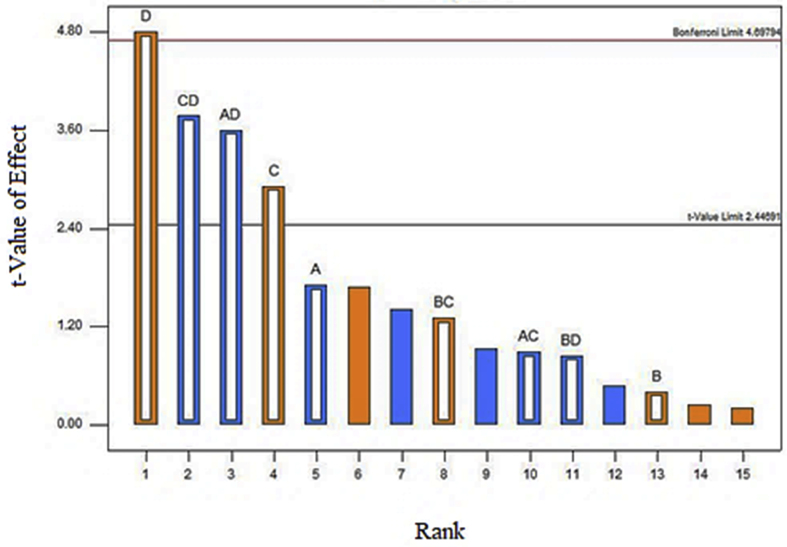
Pareto Chart of COD removal (%).

The Pareto chart displayed the main and interaction effects of the factors for the removal of COD in Figure 2. From the chart, retention time and agitation exhibit the significance in the removal of COD as the main effect. Meanwhile, SW: AMC ratio and support media displayed an insignificant effect on COD removal since the bar lies below the t-value limit. As illustrated in Table 6, retention time exhibited the highest percent contribution among other factors towards COD removal. As shown by Figure 2, retention time and agitation presented a positive influence on the removal of COD. The percentage of COD removal is increasing with increased retention time and agitation speed. When the factor is proportional to the response value, the effect is positive. Musa and Idrus (2020) stated that the percent removal of COD in wastewater rose from 92% to 96% after changing the retention time from 1 day to 3 days. The higher number in retention time is related to the higher COD removal percentage efficiency. Nayl et al. (2017) also proved that the higher agitation rate resulted in higher COD removal for municipal wastewater. Halalsheh et al. (2011) reported that the longer retention time in wastewater contributes to the scum's reduction, developing the potential of a system and greater removal of COD. As for the effects of interaction, two interaction effects of CD (agitation and retention time) and AD (SW: AMC ratio and retention time) contributed to the removal of COD with the negative effects.

**Figure 3:**
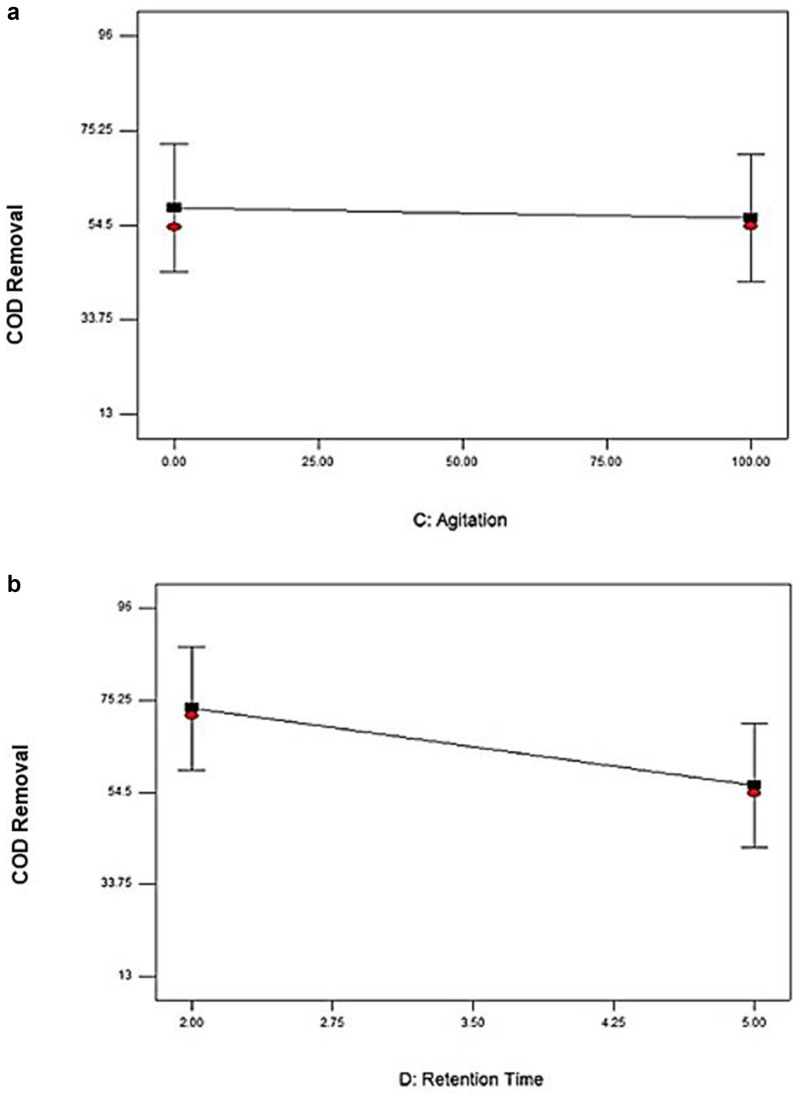
Effect of the most effective independent factors on COD removal (%) (a) Agitation (b) Retention time.

**Figure 4:**
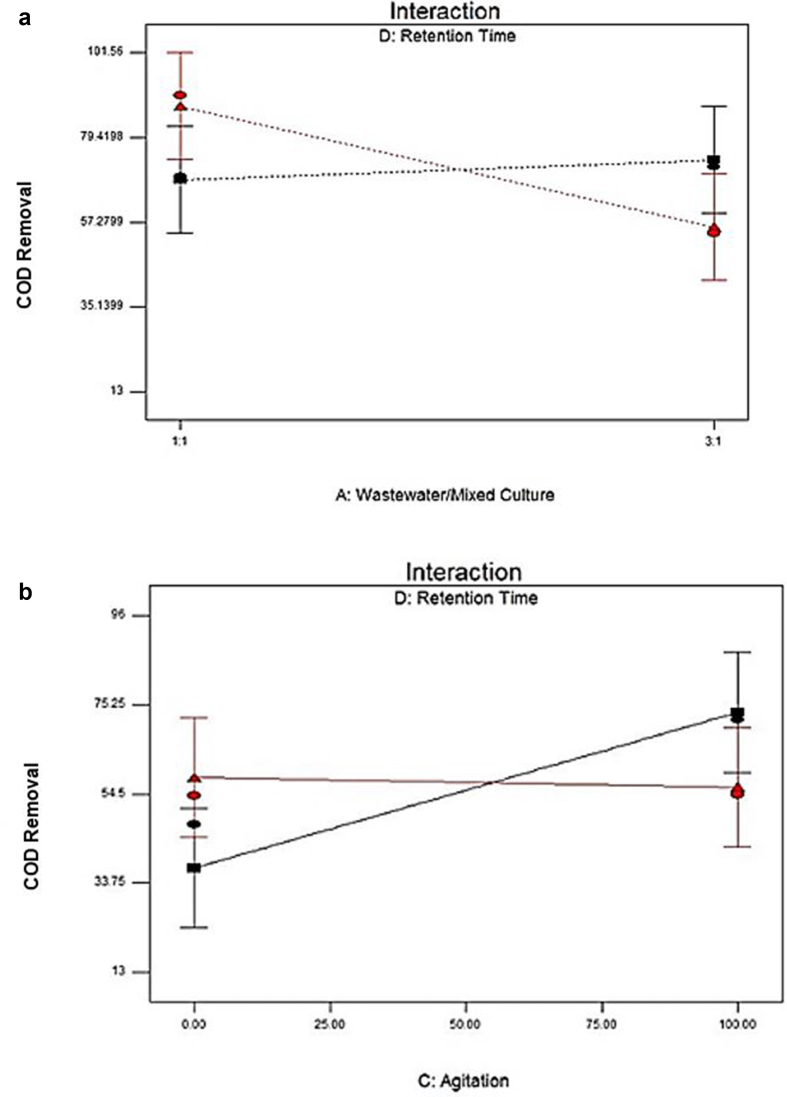
Analysis of interaction effects on COD removal (%) (a) Factor ratio SW: AMC and retention time (AD) (b) Factor agitation and retention time (CD).

Figure 3 represented the effect of two independent variables on the removal of COD. Figure 3a shows the agitation value for 0 rpm and 100 rpm. The percent removal of COD was 58.32% at 0 rpm and 56.04% at 100 rpm, which slightly lower than at 0 rpm agitation speed. These implied the insignificant impact both agitation values shown of the removal of COD. Hu et al. (2019) revealed that the cultivation of *P. cruentum* was optimum at 150 rpm agitation speed, with the increase of its biomass concentration approximately 19–57% compared to the cultivation of microalgae at other agitation speeds. From Figure 3b, the removal of COD was up to 73.46% with 2 days of retention times. At 5 days of retention times, however, the value decreased to 56.04%. Short retention time maximizes COD removal. Jiang et al. (2020) stated that the extension of hydraulic retention to 30 hours caused the decreased biomass, which indicated the discharge of the poor adaptability of microorganism and low microbial activity in the reaction system gradually. The microbial community structure was no longer suitable for the current operating conditions (Li et al., 2009; Kurokawa and Tanisho, 2005) when hydraulic retention time extended to 40 hours; the possible reason could be due to the continuous loss of biomass. The removal rates of COD and TSS could be optimized by designing the comparatively shorter hydraulic time and optimizing the effectiveness of COD removal in municipal wastewater (Christianson et al., 2016). Figure 4a and b presented the effect of an interaction between the ratio SW: AMC and retention time and between SW: agitation and retention time on COD removal.

### 3.4 Factor screening for ammonia-N removal

The percent contribution of each factor towards the removal of ammonia-N was A – Ratio SW:AMC (7.20%), B – Support Media (15.67%), C – Agitation (1.33%) and D – Retention Time (3.06%). The support media has the highest percentage contribution and, the agitation has the least percentage contribution. The highest removal of ammonia-N was noted on day 2 of retention time which is 66.67%. Table 7 summarizes the ANOVA for ammonia-N removal. The R^2^ of 0.9831 implied that the process could be represented by this model.

**Table 7:** ANOVA table for ammonia-N removal (%).

Source	Sum of squares	Df	Mean squares	F value	P-value
Model	3673.30	12	306.11	14.55	0.0245
A	269.04	1	269.04	12.79	0.0374
B	585.52	1	585.52	27.83	0.0133
C	49.74	1	49.74	2.36	0.2218
D	114.22	1	114.22	5.43	0.1022
AB	116.69	1	116.69	5.55	0.0999
AC	63.16	1	63.16	3.00	0.1816
AD	499.41	1	499.41	23.73	0.0165
BC	982.35	1	982.35	46.69	0.0064
BD	63.16	1	63.16	3.00	0.1816
CD	116.69	1	116.69	5.55	0.0999
ACD	585.52	1	585.52	27.83	0.0133
BCD	227.78	1	227.78	10.83	0.0461

**Figure 5:**
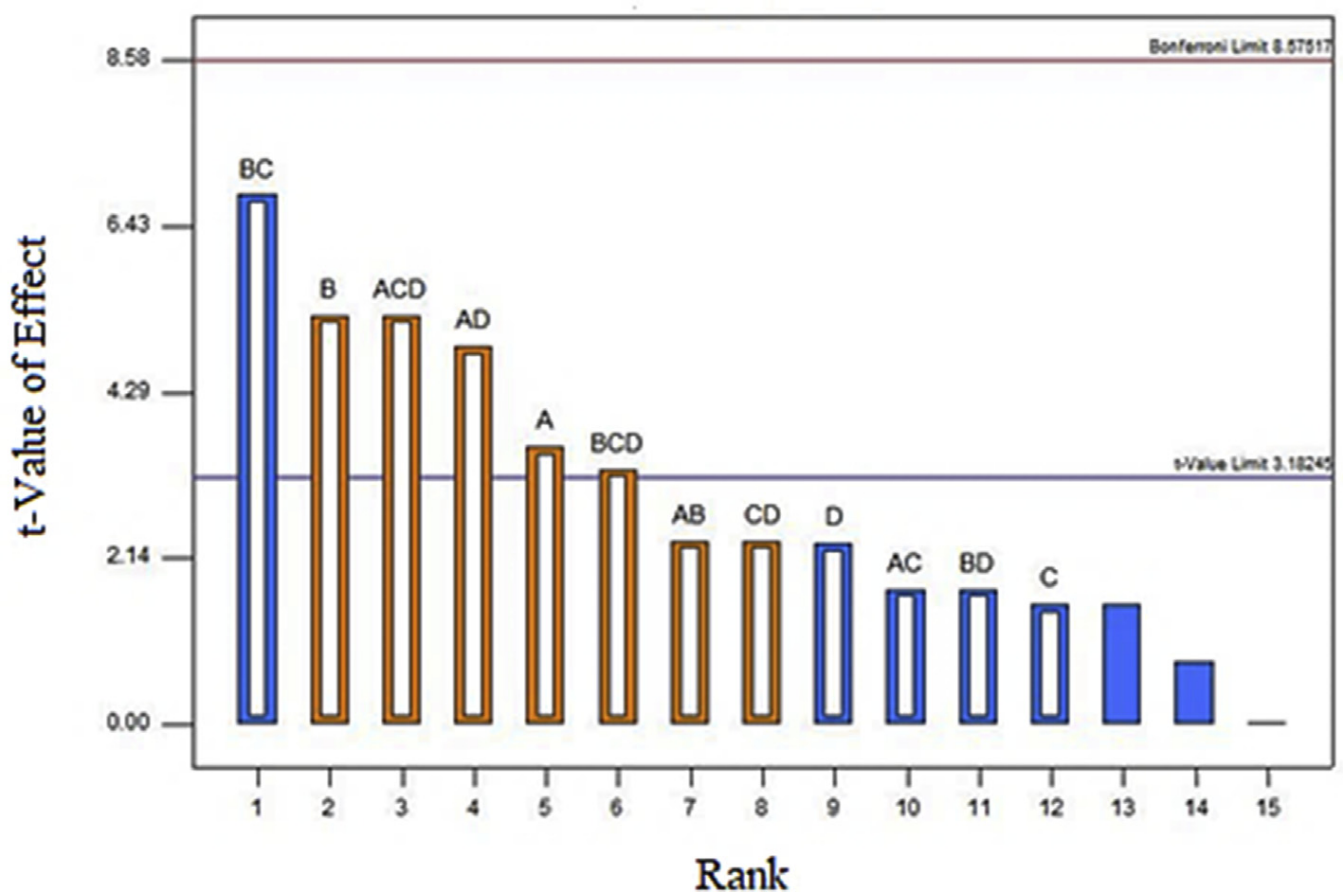
Pareto Chart of ammonia-N removal (%).

Figure 5 displayed the Pareto chart, showing the main and interaction effects of the factors on ammonia-N removal. For the main effect, support media exhibited the highest percentage contribution to ammonia-N removal. Chung et al. (2000) proved that support media mostly effective in removing ammonia-N in wastewater such as zeolites. There was approximately 88–92% removal of ammonia-N and high settleability in the final discharge of wastewater in this experiment. As exhibited by Table 8, support media contributed the most to the removal of ammonia-N compared to other factors. Figure 5 shows the positive effect of support media on the removal of ammonia-N. Valdivia et al. (2007) proved that using polypropylene foam as support media, ammonia removal values could be achieved over 50% from municipal wastewater. For the effects of interaction, factor BC (support media and agitation) contributed the most to the removal of ammonia-N, followed by factor AD (SW: AMC ratio and retention time). The experiments conducted by Martins et al. (2018) concluded that 100 rpm of agitation speed was the best condition among other parameters that lowered ammonia-N concentration in wastewater.

**Table 8:** Suggested best conditions of COD and ammonia-N for municipal wastewater.

Factor	Range
Ratio SW: AMC	3:1
Support Media	No
Agitation (rpm)	100
Retention time (days)	5

**Figure 6:**
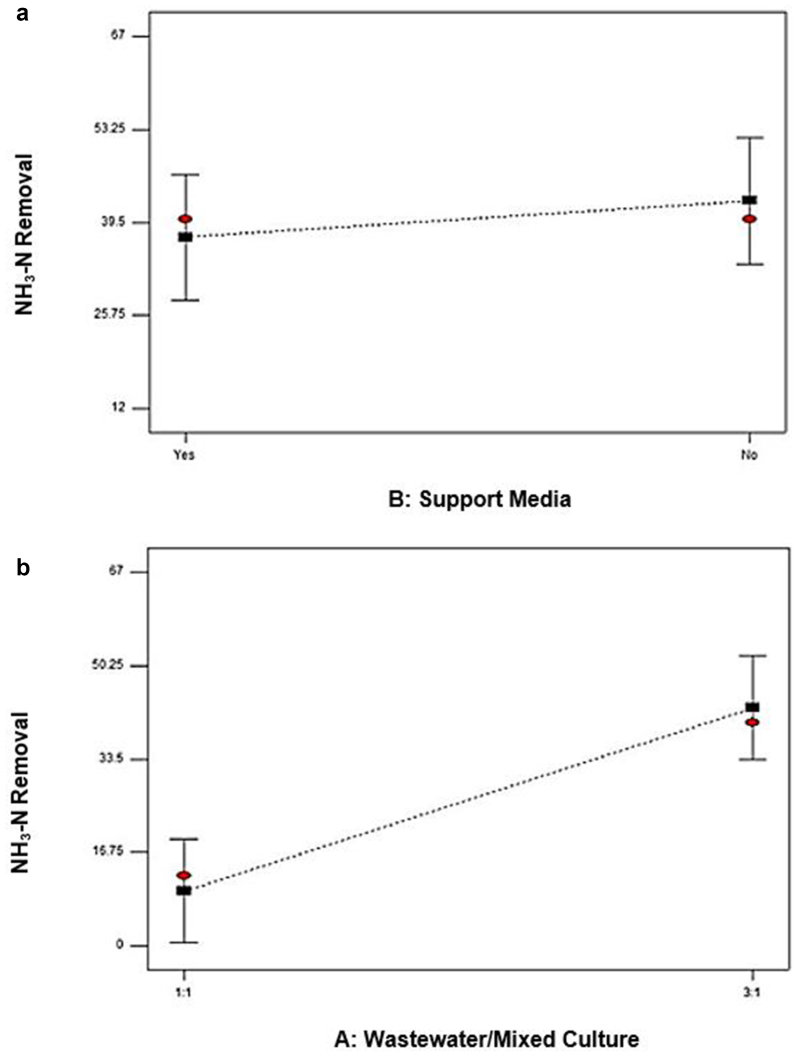
Most effective independent factor in ammonia-N removal (%) (a) Factor support media (b) Factor ratio SW: AMC.

Figure 6 exhibited the effect of two independent variables on the removal of ammonia-N. As illustrated by the Pareto chart in Figure 5, the support media displayed as the most significant factor in removing ammonia-N as it lies above the t-value limit line in a positive effect. From Figure 6a, Ammonia-N removal reached up to 37.30% when support media was used; however, the value of ammonia-N removal increased to 42.70% without support media. In the wastewater industry, many smaller treatment sewages that works do exist, which faced the discharge limits of ammonia but, where denitrification is non-essential. This was proven that sometimes, ammonia-N removal works better even without support media (Horan, 1994). Figure 6b shows that the studied ratio of SW: AMC (1:1 and 3:1) is significant to ammonia-N removal. Ammonia-N removal was 9.80 % at a ratio of 1:1 while it became higher, which was 42.70 % when the ratio of 3:1 was applied. The removal rate of ammonia-N was two times higher in wastewater treatment with the application of mixed culture rather than a single culture and that the ratio of denitrification was remarkably high as compared to in single culture (Joo et al., 2007).

**Figure 7:**
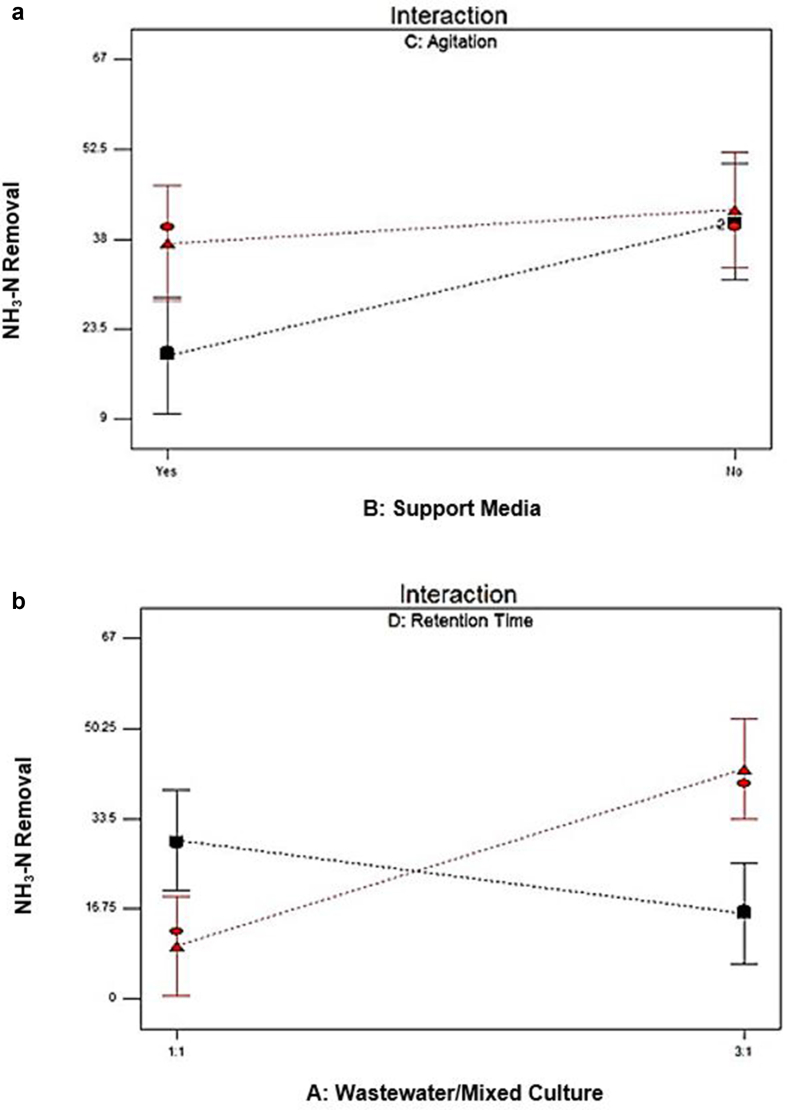
Interaction factor for ammonia-N removal (%) (a) Factor support media and retention time (BC) (b) Factor ratio SW: AMC and retention time (AD).

Figure 7a presented the effect of an interaction between the support media and agitation and between SW: AMC (1:1 and 3:1) ratios and retention time (2 and 5 days) on the removal of ammonia-N. Good performance was seen on the removal of ammonia-N when 0 rpm was applied (black line). The significant difference in the presence of support media was exhibited on ammonia-N removal, as the removal was up to 40.83% without the support media, and only 19.17% removal was achieved with the presence of support media. These implied that without agitation, the removal rates were better than with the involvement of support media because support media can be a disturbance for the ammonia-N removal when agitation is not applied (Sonawane et al., 2015). A better performance was observed when 100 rpm (red line) agitation speed was applied than 0 rpm. The removal rates of ammonia-N exhibited no significant difference with or without the presence of support media, with 37.30% and 42.70%, respectively. The increment in ammonia-N removal at 100 rpm was likely because of two possibilities. First was the presence of microorganisms associated with Pseudomonas sp. and Comamonas sp. in the bed reactor that caused the endogenous denitrification of biomass lysed by shearing at that agitation speed. The lack of Ar/CO_2_ atmosphere in the headspace of the reactor to sustain the anaerobic agitation conditions at that speed was also one of the possibilities. This condition had caused the enabling of nitrification by nitrite-oxidizing bacteria (NOB) and subsequent reduction of nitrate because at 100 rpm there was no more nitrate accumulation than the one produced by the anaerobic oxidation of ammonium (Martins et al., 2018). As designated by the Pareto chart in Figure 5, support media was observed as the most significant factor as the bar extends above the t-value limit and displayed positive values.

From Figure 7b, when 2 days of retention time (black line) was used, the performance on ammonia-N improved at SW: AMC ratio of 1:1, which is 29.44%, while dropped to 15.80% at 3:1 ratio. These are because even at a short time (2 days), the same amount of the reaction between synthetic wastewater and acclimatized mixed culture can already be completed. The significant difference was perceived when 5 days of retention time (black line) was applied at both ratios and produced better performance on ammonia-N removal. At a ratio of 1:1 SW: AMC, ammonia-N removal was only 9.80 %, while at 3:1 ratio, the ammonia-N removal presented a higher value which was 42.70 %. The results indicate that retention time plays an essential role in ammonia removal. Faizal et al. (2014) also agreed that biological treatment occurs with a retention time of 63–250 minutes, and the range of initial concentration of ammonia-N from 276 to 530 mg/L gives higher ammonia-N removal from 20.92 – 56.52% in wastewater treatment.

### 3.5 Best conditions for COD and ammonia-N removal

A validation experiment was carried out based on the best conditions suggested by Design-Expert software (Table 8) by using a SW: AMC ratio of 3:1, an agitation speed of 100 rpm, and a retention time of 5 days. At those parameters, COD and ammonia-N removal were at the highest percentage. El-Gawad and El-Aziz (2018) proven that using 100 rpm for agitation speed with 4 days of retention time resulted in 67% of COD removal in wastewater. Adeleke et al. (2019) also revealed that better performance of COD removal in wastewater was obtained by using 150 agitation speeds which is up to 71%. The experiment was carried out in three runs to calculate the percentage error between the actual and predicted result using Eq. (2). Table 9 presented the predicted and actual value for COD and ammonia-N removal. Run 2 was selected due to the lowest value of error for COD and ammonia-N compared to other runs. The low value of error indicated that the experimental value was reasonably closed to the predicted values. From the validation experiment, we managed to get only 57.23% for COD removal and 43.20% ammonia-N removal, respectively.

(2) $$Error\;\left(\%\right)=\frac{Experimental-Predicted}{Predicted}\times 100$$

**Table 9:** Validation study for COD and ammonia-N removal.

Run	COD Removal (%)			Ammonia-N Removal (%)		
	P	A	E	P	A	E
1	56.04	58.46	4.14	42.71	43.99	2.91
2		57.23	2.08		43.20	1.13
3		54.76	2.34		44.44	3.89

P: Predicted; A: Actual; E: Error.

### 3.6 Optimization study of COD removal

The best conditions obtained from TLFD screening were optimized by RSM method. In this design of experiment, CCD was implemented for the optimization of COD and ammonia-N removal. The two major factors involved in this study were agitation speed and retention time. A total of 13 experimental runs were generated with different setup conditions (Table 10). A second-order quadratic equation was established with the experimental data and was presented by Eqs. (3) and (4) in terms of coded and actual values, respectively.

(3) $$COD=\;52.56+13.10x_{1}+1.78x_{2}-1.13x_{1}x_{2}\;-2.762x_{1^{2}}-0.13x_{2^{2}}$$

(4) $$COD=\;-137.9828+1.8582x_{1}+25.0225x_{2}-0.0902x_{1}x_{2}\;-4.4160E^{-003}x_{1^{2}}-1.2451x_{2^{2}}$$

**Table 10:** Experimental layout of central composite design and its values.

Run	Factors		Responses	
	Agitation (rpm)	Retention Time (days)	Ammonia-N mg/L	COD mg/L
1	75	4.5	21.43	24.94
2	125	4.5	46.43	54.96
3	75	5.5	28.57	31.61
4	125	5.5	53.57	57.12
5	50	5	14.29	19.77
6	150	5	60.71	70.6
7	100	4	28.57	51.86
8	100	6	57.14	58.1
9	100	5	46.43	55.71
10	100	5	46.43	55.19
11	100	5	50	55.61
12	100	5	50	55.71
13	100	5	50	55.24

The statistical significance of the regression model was checked by the F-test in the analysis of variance (ANOVA). Effects with a confidence level higher than 95 % (p-value less than 0.05) were preferable to represent the reliability of a result. From the ANOVA result summarized in Table 11, the p-value of the model was 0.0042, which is more than a 95% confidence level. The small p-value indicates the significance of the corresponding coefficient (Zhao et al., 2011). The agitation displayed a significant effect on the biological treatment of municipal wastewater, supported by its low p-value. The R^2^ value of 0.8781 and the R_adj_ value of 0.7911 implying that the computed model satisfactorily correspond to the experimental data. The R^2^ was used to signify how close the data to the regression line, and the value of R^2^ should not be less than 80% (Karazhiyan et al., 2011).

**Table 11:** ANOVA for response surface model for COD.

Source of Variation	Sum of Squares	df	Mean Square	F value	p-value Prob > F
					
Model	2282.10	5	456.42	10.09	0.0042
A-Agitation	2059.06	1	2059.06	45.50	0.0003
B-Retention Time	37.84	1	37.84	0.84	0.3909
Residual	316.76	7	45.25		
Lack of Fit	316.50	3	105.50	1600.43	<0.0001
Correlation Total	2598.86	12			
R^2^	0.8781				
Adjusted R^2^	0.7911				

**Figure 8:**
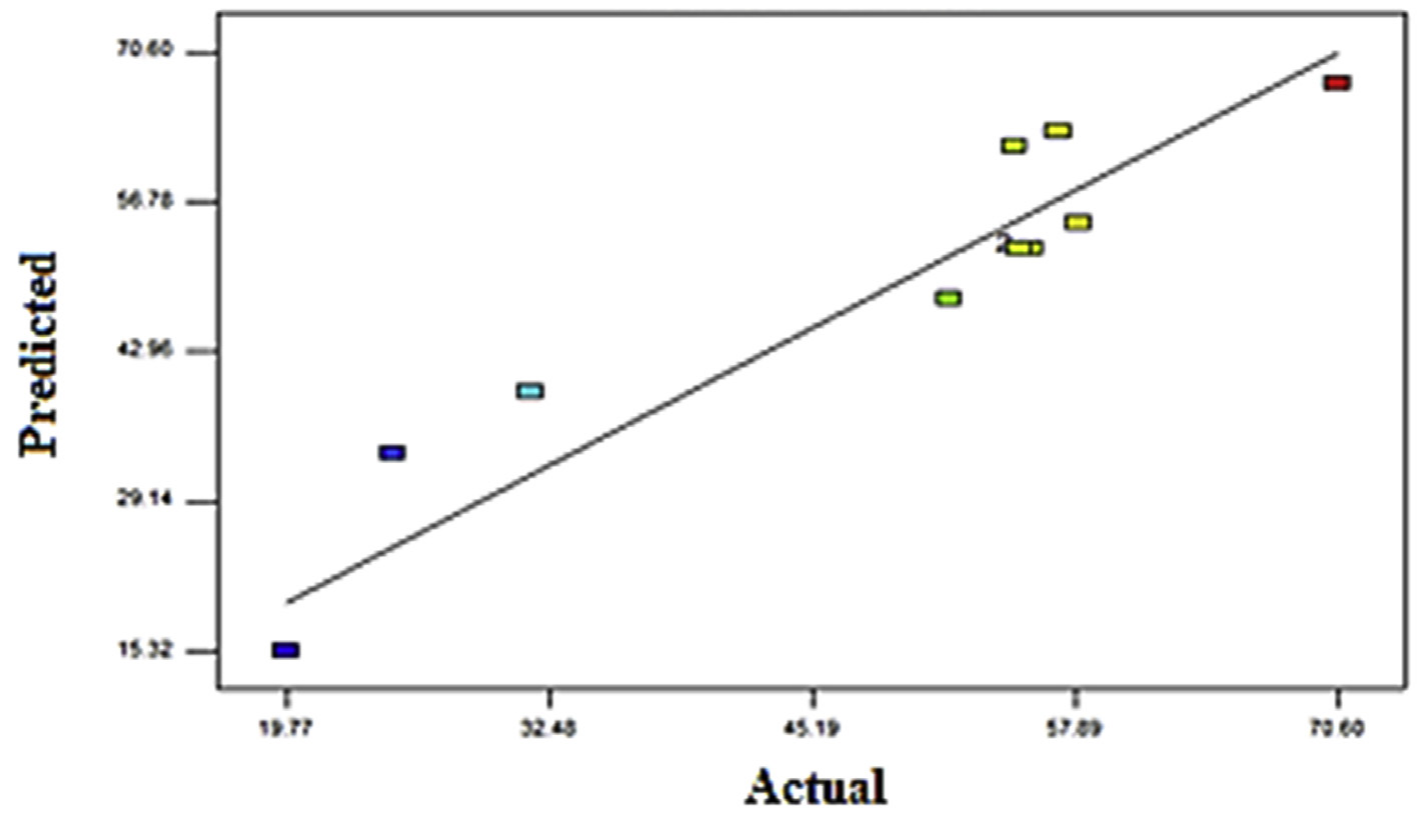
Correlation of the predicted and actual values.

**Figure 9:**
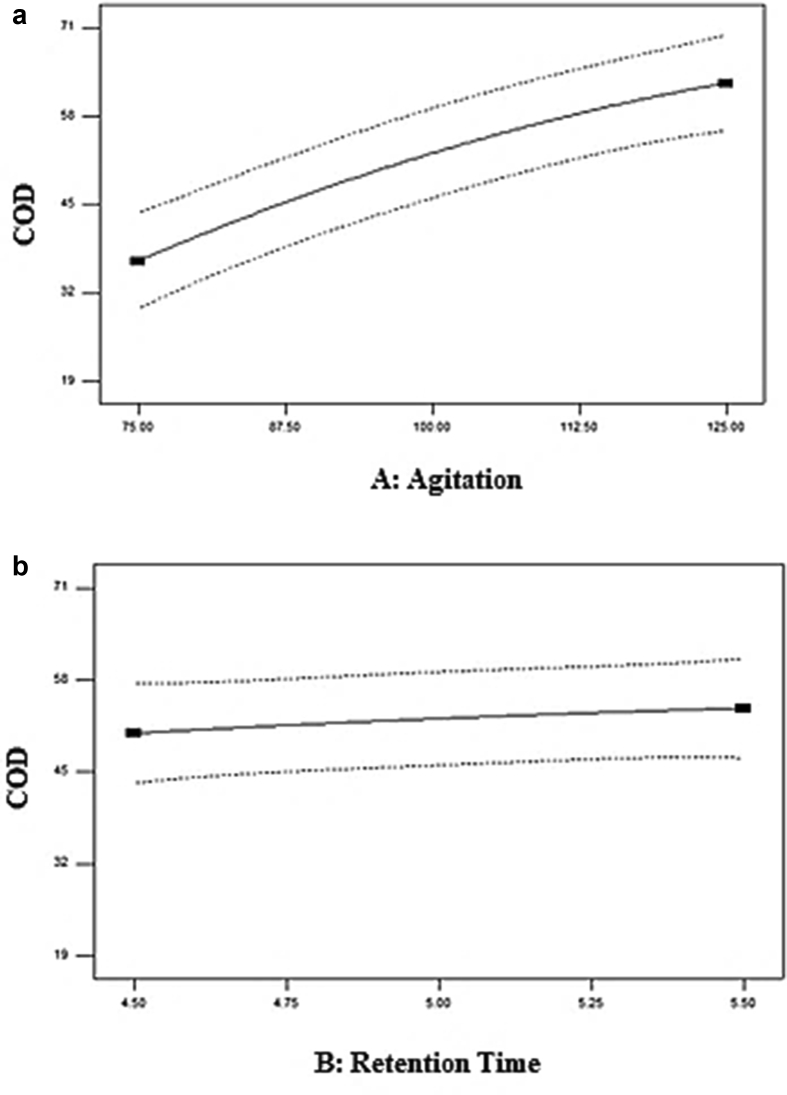
The effect of (a) agitation and (b) retention time on COD removal (%).

The correlation of actual and predicted values of COD concentration was displayed in Figure 8. A linear distribution was seen, indicating the well-fitting model. The normal probability plot reveals that the residuals followed a normal distribution, forming an almost straight line. Figure 9 displayed the influence of the two independent parameters on the COD removal. COD removal increases with agitation from 75 rpm to 125 rpm, as depicted by Figure 9(a). These indicate that the increase of agitation speed has a positive influence on COD removal. Alam et al. (2003) recorded the maximum reduction of organic substances in municipal wastewater treatment plants using a mixed culture of *Penicillium corylophilum* and *Aspergillus niger* in a batch fermenter was observed at 150 to 200 rpm agitation speed. There were no effective outcomes discovered at a higher agitation rate. Higher agitation at 120 rpm can supply adequate mixing, enhancing substrate conversion efficiency in an anaerobic digester by providing intimate contact between wastewater and its mixed culture. However, too high agitation (over 120 rpm) will cause cell disruption of mixed culture.

**Figure 10:**
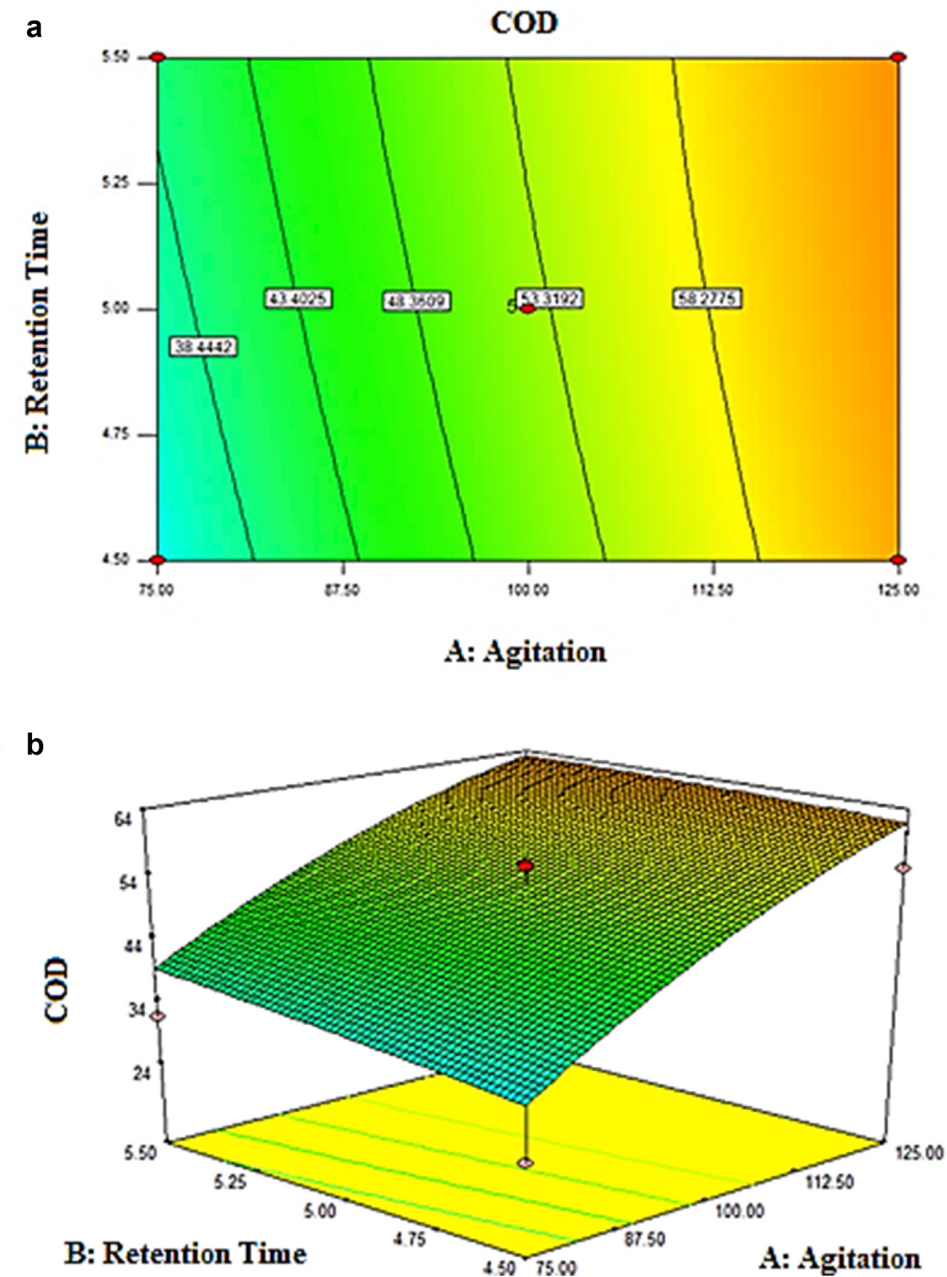
(a) Contour plot, (b) 3D plot of COD removal (%).

Figure 9(b) displayed the effect of retention time on COD removal. The figure demonstrated that the optimum retention time of COD removal was 5 days. The retention time did not contribute much to the COD removal as there was only a slight difference between the COD removal from 4.5 to 5 days. These explained the large p-value of 0.3909 obtained from the ANOVA (Table 11). The interaction effect among parameters on COD removal was represented graphically by the contour and 3D plot, portrayed in Figure 10a and b. The figures demonstrated that the COD removal at any retention time was influenced by the agitation speed. The increase in agitation speed had caused an increase in COD removal. The optimum conditions for COD removal value of 70.6%% were 150 rpm and 5 days of retention time.

### 3.7 Optimization study of Ammonia-N removal

Thirteen experimental runs with different retention times and agitation speeds were performed according to Table 10. A second-order polynomial equation was developed with the experimental data and was presented in both coded and actual forms displayed by Eqs. (5) and (6), respectively.

(5) $$Ammonia-N=\;46.68\;+11.90x_{1}+5.95x_{2}+0x_{1}x_{2}\;-2.98x_{1^{2}}-1.55x_{2^{2}}$$

(6) $$Ammonia-N=\;-261.417+1.3998x_{1}+73.8145x_{2}-4.978E^{-015}x_{1}x_{2}\;-4.6185E^{-003}x_{1^{2}}\;-6.1911x_{2^{2}}$$

The ammonia-N removal model was better fitted with a quadratic polynomial model since the adjusted R2 of the quadratic model (0.8759) was higher than other models for all responses. The ANOVA in Table 12 showed the p*-*value of the model was 0.0007, which is significant. The p-value below 0.05 indicates the significancies of the model. The agitation displayed a significant influence on the removal of ammonia-N, supported by its less than 0.0001 p-values. The R^2^ value of 0.9276 and the R_adj_ value of 0.8759 signified that the computed model and the experimental data were adequately matched. Olmez (2009) proposed that for a good fit of a bioprocess model, R^2^ should at least be 0.80. Since the R^2^ for this model is more than 0.8, this model was accepted and could present the process.

**Table 12:** ANOVA for response surface model for ammonia-N removal (%).

Source of Variation	Sum of Squares	df	Mean Square	F-value	p-value Prob > F
Model	2329.47	5	465.89	17.93	0.0007
A-Agitation	1700.27	1	1700.27	65.45	<0.0001
B-Retention Time	425.07	1	425.07	16.36	0.0049
AB	0	1	0	0	1.0000
A^2^	190.92	1	190.92	7.35	0.0302
B^2^	54.89	1	54.89	2.11	0.1894
Residual	181.84	7	25.98		
Lack of Fit	166.55	3	55.52	14.52	0.0129
Correlation Total	2511.31	12			
R^2^	0.9276				
Adjusted R^2^	0.8759				

**Figure 11:**
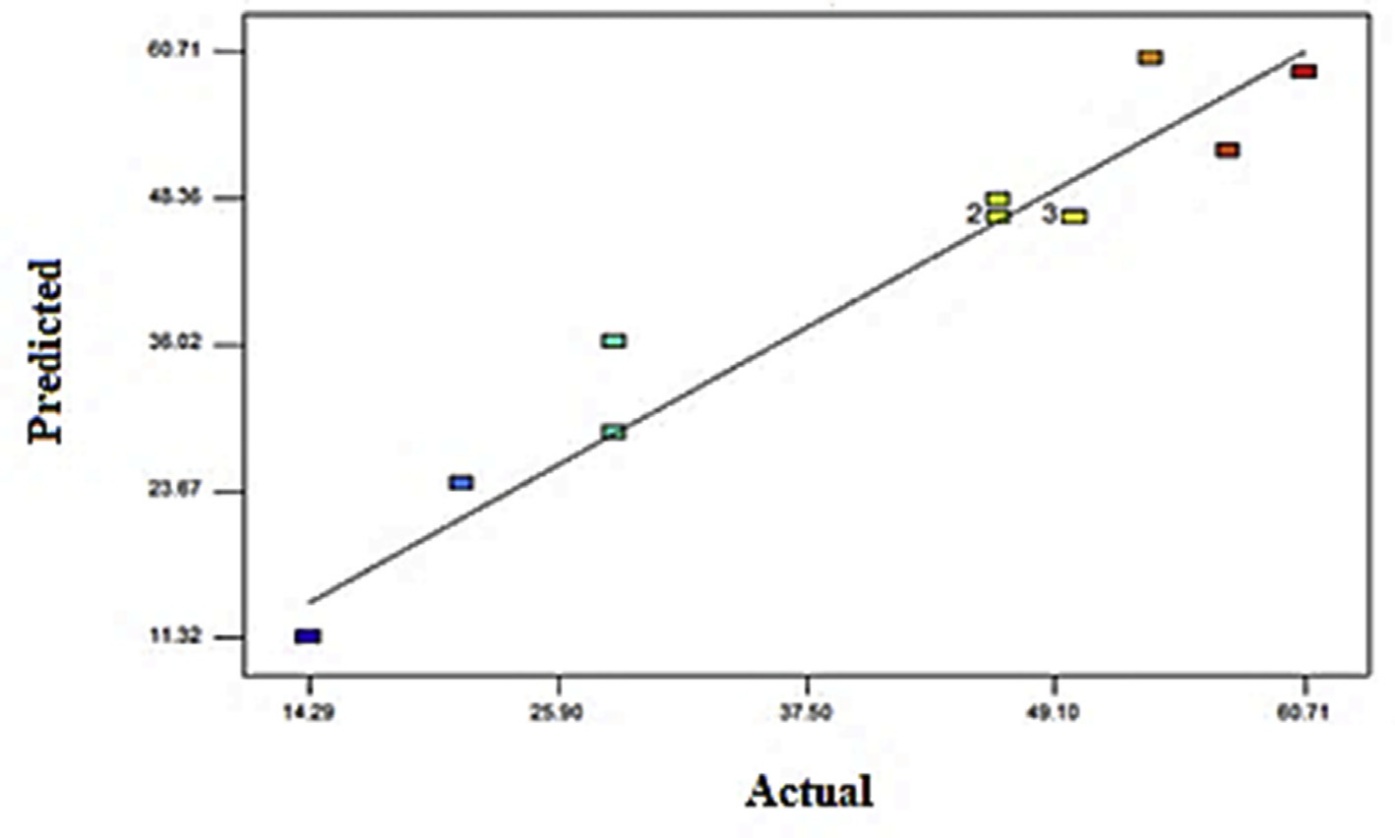
Correlation of the predicted and actual values.

**Figure 12:**
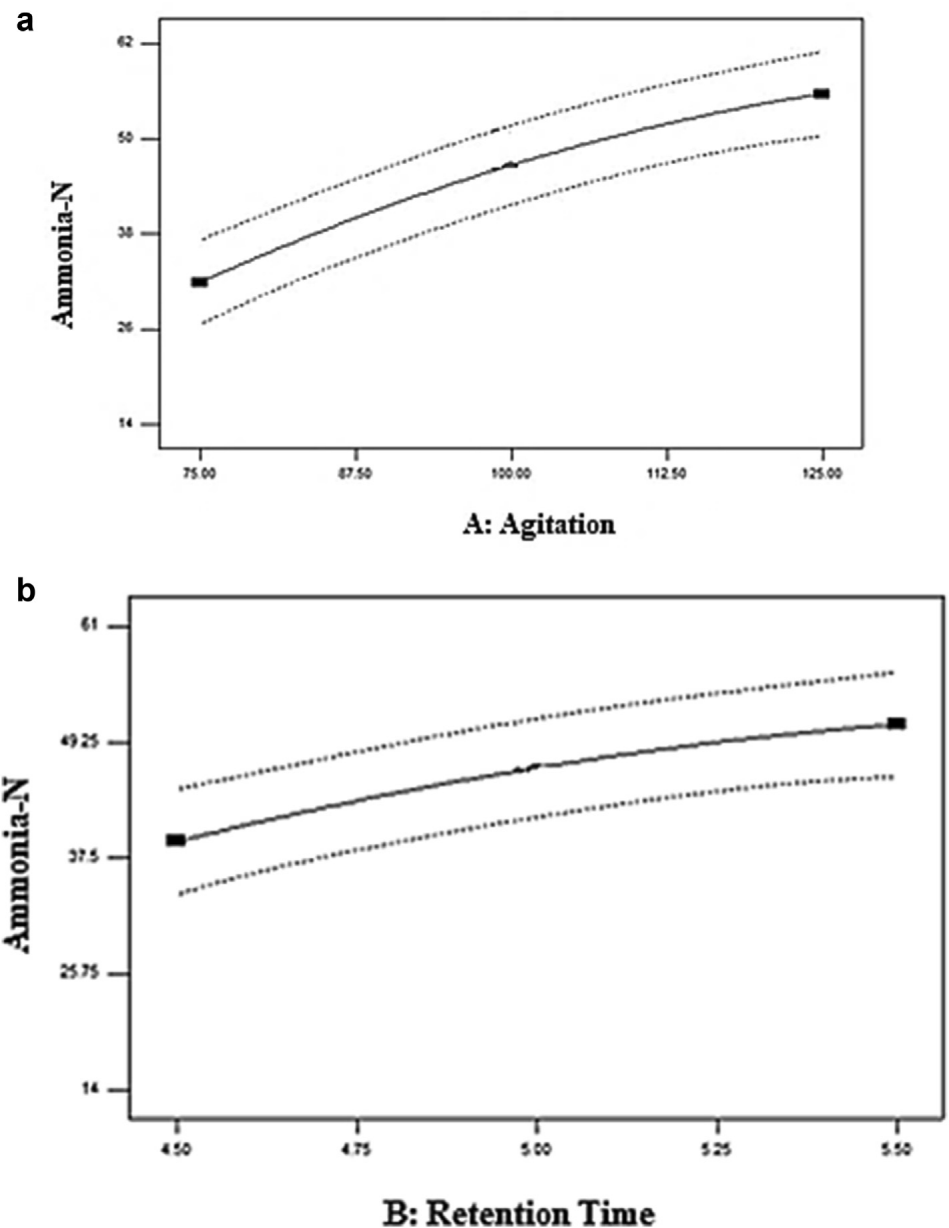
Effect of (a) agitation and (b) retention time on ammonia-N removal (%).

Figure 11 portrayed the correlation of predicted and actual values of ammonia–N concentration displaying a linear dispersion. These suggest that the model was well-fitted with the expected values comparable with the observed values. Figure 12a and b depicted the influence of independent parameters on ammonia-N removal. The plotted data demonstrate the effects of agitation and retention times on ammonia-N removal. Both parameters were observed to be significant contributors to ammonia-N removal. Ammonia-N removal increased from 28.57 to 53.57 percent, with an increase of agitation from 75 rpm to 125 rpm, as shown in Figure 5a. The effect of retention time was displayed in Figure 5b. The optimum retention time for the ammonia-N removal is at 5 days, with 60.71% removal.

The agitation speed recorded by Alam et al. (2003) shows that the maximum reduction of organic substances in oil palm industrial wastes was obtained at 150–200 rpm. A higher reduction (3%) of organic material was obtained in the fungal-treated system. There were no effective results discovered at an agitation speed of more than 200 rpm. Therefore, 150 rpm was selected as the optimized mixing speed and made constant during the treatment process. While for retention time, longer retention time led to an increased ammonia-N removal percentage as the reaction between the microbes and the wastewater was completed (Gil et al., 2011). These were also proved by the shaking speed and retention time recorded by Lalung and Ismail (2015), with 150 rpm of shaking speed and 5 days of retention time for reducing the organic matter by bacterial strains in palm oil mill effluent.

**Figure 13:**
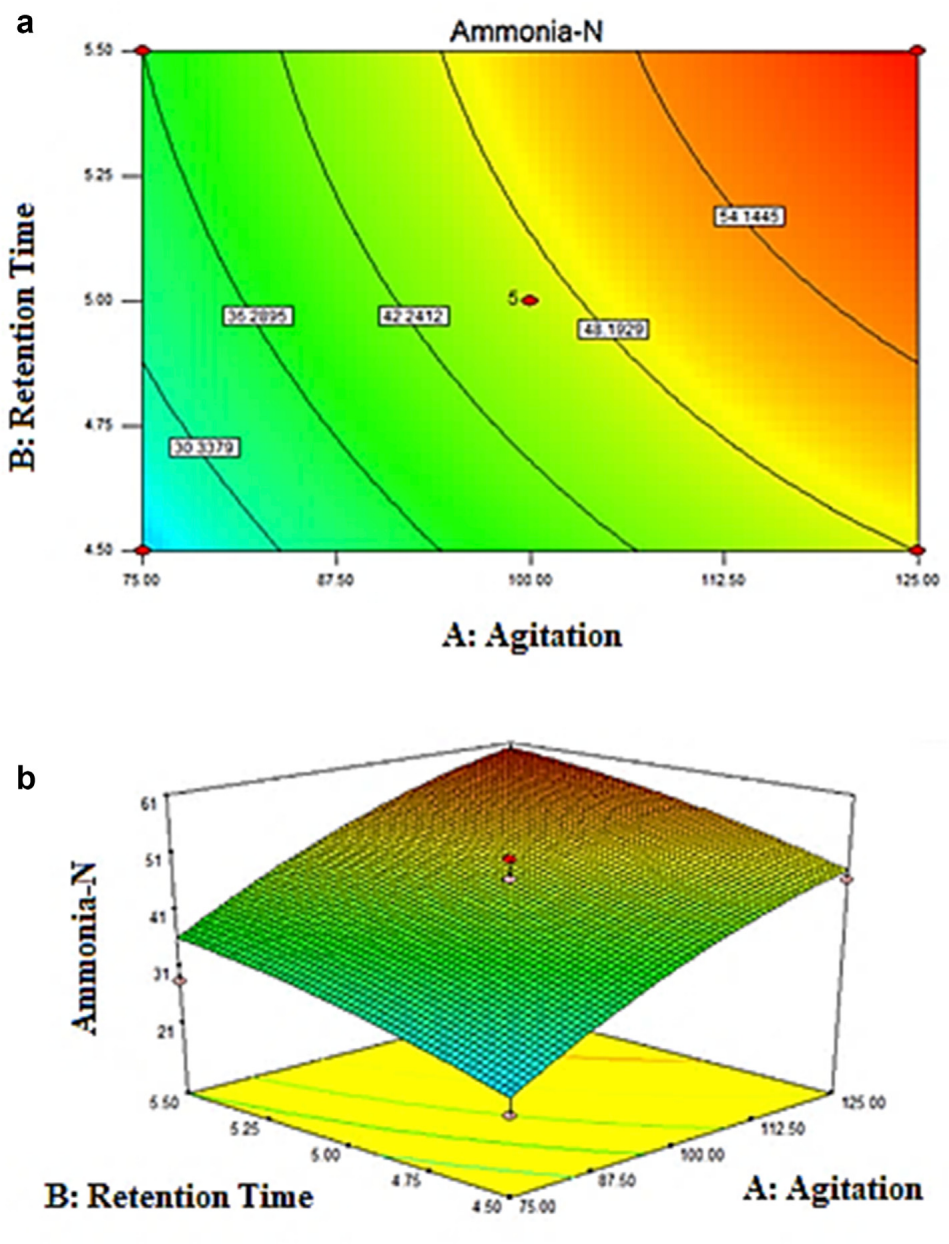
(a) Contour plot (b) 3D plot of ammonia-N removal (%).

The interaction between retention time and agitation speed on the removal of ammonia-N was demonstrated by the contour and the 3D plot (Figure 13a and b). Increasing agitation resulted in the higher removal of ammonia-N. Figure 6a clearly showed that at the agitation of 150 rpm and retention time of 5 days, ammonia-N had the highest removal. From the contour plot, the elliptical profile proved an interaction between agitation and retention time. It can be explained that as agitation increased, the removal of ammonia-N was increased. Nevertheless, once the agitation and retention time was greater than the center point value, the reverse trend was observed. From Figure 6b, the 3D surface graph generated in a slight dome shape in which maximum points were obtained at standard Run 6 whose removal of ammonia-N was 60.71 percent.

### 3.8 Optimum conditions

Table 13 represented the optimum conditions suggested by the Design-Expert software and from the validation experiment. It was observed that 60.1% of ammonia-N removal and 64.6% of COD removal were achieved at 125 rpm and 5.5 days. The suggested optimum condition obtained from the experiment reported that the optimum condition of 150 rpm agitation speed and 5 days of retention times had resulted in 70.6% and 64.6% of COD and ammonia-N removal, respectively. The validation experiment was conducted according to the two different suggested optimum conditions to validate the results. The outcome shows that the validation values were reasonably comparable with the predicted values. The removal of ammonia-N and COD of 64.29% at 70.41% was obtained at optimum conditions of 150 rpm and 5 days of retention times.

**Table 13:** Predicted and validation experiment of COD and ammonia-N removal (%).

Factors	Value	Value
A: Agitation (rpm)	125	150
B: Retention time (days)	5.5	5
Predicted Ammonia-N removal (%)	60.1	60.71
Predicted COD removal (%)	64.6	70.6
Experimental Ammonia-N removal (%)	57.14	64.29
Experimental COD removal (%)	64.73	70.41
Error Ammonia-N removal (%)	5.17	5.56
Error COD removal (%)	0.19	0.27

A similar study by Nayl et al. (2017) had proved that a higher agitation speed applied had resulted in higher COD (90.4%) and ammonia-N (80.6%) being removed from the wastewater with 100–700 rpm agitation speed. Wang et al. (2016) stated that the concentration of COD increased with low agitation speed and displaying an opposite tendency with rapid mixing speed though there still were some fluctuations. It could be deduced that moderate agitation by slow mixing could help fulfilling collision and contact when combined with the morphology of flocks during experiments. Slight mixing suppressed the process; intense mixing had an adverse effect due to its irreversible damage to flocks.

Short retention time maximizes COD and ammonia-N removal. Jiang et al. (2020) stated that when retention time was extended to 30 hours, the biomass began to decrease, which indicated that the microorganisms with poor adaptability and low microbial activity in the reaction system were gradually discharged. The microbial community structure was no longer suitable for the current operating conditions (Li et al., 2009; Kurokawa and Tanisho 2005) when retention time extended to 40 hours; the possible reason could be the continuous loss of biomass. Designing for relatively shorter retention time optimized COD and ammonia-N removal rates (Christianson et al., 2016). However, according to Li et al. (2014), the removal percentages of COD and ammonia-N were increased with the increase of retention time from 5 to 40 days. These illustrate that the longer retention time had resulted in more COD and ammonia-N being removed from the systems. The result obtained from the previous study was reported to be similar to the present study. The percentage of error was predicted using Eq. (2). The error was ranging from 0.19% to 5.56%.

## 4 Conclusion

This study was conducted objectively to determine the most significant factors contributing to the removal of COD and ammonia-N from a municipal wastewater treatment plant by using two-level factorial analysis. The best conditions for wastewater from the municipal wastewater industry were a SW: AMC ratio of 3:1, 100 rpm agitation, no support media and a retention time of 5 days. COD and ammonia-N removal percentage achieved up to 57.23% and 43.20%. The major factors contributing to the inhibition of microbial growth were further optimized through a response surface methodology. The maximum COD and ammonia-N removal achieved was 70.41% and 64.29% at optimum conditions of 150 rpm and 5 days. These results show that the COD and ammonia-N removal was increased from 57.23% to 70.41% for COD and 43.20% to 64.29% for ammonia-N.

## Declarations

### Author contribution statement

Norazwina Zainol: Conceived and designed the experiments; Contributed reagents, materials, analysis tools or data.

Amirah Ya'acob & Nor Hazwani Aziz: Performed the experiments; Analyzed and interpreted the data; Wrote the paper.

### Funding statement

Dr. Norazwina Zainol was supported by 10.13039/501100005605Universiti Malaysia Pahang [RDU210341].

### Data availability statement

Data included in article/supp. material/referenced in article.

### Declaration of interests statement

The authors declare no conflict of interest.

### Additional information

No additional information is available for this paper.

## References

[bib1] Adeleke O.A., Latiff A.A.A., Saphira M.R., Daud Z., Ismail N., Ahsan A., Aziz N.A.A., Al-Gheethi A., Kumar V., Fadilat A., Apandi N., Ahsan A., Ismail A.F. (2019). Nanotechnology in Water and Wastewater Treatment: Theory and Applications.

[bib2] Alam M.Z., Razi A.F., Aziz S.A., Molla A.H. (2003). Optimization of compatible mixed cultures for liquid. Water, air and soil pollution. Methods Appl..

[bib3] Belli T.J., Bernardelli J.K.B., da Costa R.E., Bassin J.P., Amaral M.C.S., Lapolli F.R. (2017). Effect of solids retention time on nitrogen and phosphorus removal from municipal wastewater in a sequencing batch membrane bioreactor. Environ. Technol..

[bib4] Christianson L.E., Lepine C., Sharrer K.L., Summerfelt S.T. (2016). Denitrifying bioreactor clogging potential during wastewater treatment. Water Res..

[bib5] Chung Y.C., Son D.H., Ahn D.H. (2000). Nitrogen and organics removal from industrial wastewater using natural zeolite media. Water Sci. Technol..

[bib6] Constanza C.M., Roberto E.D., Lisette H.E., Laura R.C., Bárbara B.S., Flavia D., Michael S. (2019).

[bib7] Devi R., Singh V., Kumar A. (2008). COD and BOD reduction from coffee processing wastewater using Avacado peel carbon. Bioresour. Technol..

[bib8] El-Gawad S.A.A., El-Aziz H.M.A. (2018). Effective removal of chemical oxygen demand and phosphates from aqueous medium using entrapped activated carbon in alginate. Moj Biol. Med..

[bib9] Faizal M., Haryati S., Kurniawati H. (2014).

[bib10] (2017). Fluence News Team. https://www.fluencecorp.com/what-is-biological-wastewater-treatment/.

[bib11] Gil M.V., Carballo M.T., Calvo L.F. (2011). Modelling N mineralization from bovine manure and sewage sludge composts. Bioresour. Technol..

[bib12] Halalsheh M., Kassab G., Yazajeen H., Qumsieh S., Field J. (2011). Effect of increasing the surface area of primary sludge on anaerobic digestion at low temperature. Bioresour. Technol..

[bib13] Horan N.J. (1994).

[bib14] Hu H., Wang H.F., Li J.Y., Ma L.L., Shen X.F., Zeng R.J. (2019). Evaluation of the effect of agitation speed on the growth and high-value LC-PUFA formation of Porphyridium cruentum based on basic rheological analysis. J. Chem. Technol. Biotechnol..

[bib15] Jamshidnezhad M. (2015).

[bib16] Jiang F., Peng Z., Li H., Li J., Wang S. (2020). Effect of hydraulic retention time on anaerobic baffled reactor operation: enhanced biohydrogen production and enrichment of hydrogen-producing acetogens. Processes.

[bib17] Joo H.S., Hirai M., Shoda M. (2007). Improvement in ammonium removal efficiency in wastewater treatment by mixed culture of Alcaligenes faecalis No. 4 and L1. J. Biosci. Bioeng..

[bib39] Karazhiyan H, Razavi S.M.A, Phillips G.O (2011). Extraction optimization of a hydrocolloid extract from cress seed (*Lepidium sativum*) using response surface methodology. Food Hydrocolloids..

[bib18] Khalid S., Shahid M., Bibi I., Sarwar T., Shah A.H., Niazi N.K. (2018). A review of environmental contamination and health risk assessment of wastewater use for crop irrigation with a focus on low and high-income countries. Int. J. Environ. Res. Publ. Health.

[bib19] Khaliq S.J.A., Al-Busaidi A., Ahmed M., Al-Wardy M., Agrama H., Choudri B.S. (2017). The effect of municipal sewage sludge on the quality of soil and crops. Int. J. Recycl. Org. Waste Agric..

[bib20] Kurokawa T., Tanisho S. (2005). Effects of formate on fermentative hydrogen production by *Enterobacter aerogenes*. Marine. Biotechnology.

[bib21] Ladu J.L.C., Xiwu L., Meiling Z., Ting W. (2014). Application of A2/O bio-reactor & constructed wetlands for removing organic and nutrient concentrations from rural domestic sewage. Int. J. Environ. Sci..

[bib22] Li J., Zheng G., He J., Chang S., Qin Z. (2009). Hydrogen-producing capability of anaerobic activated sludge in three types of fermentations in a continuous stirred-tank reactor. Biotechnol. AdV..

[bib23] Massalha N., Plewa M.J., Nguyen T.H., Dong S. (2020). Influence of anaerobic mesophilic and thermophilic digestion on cytotoxicity of swine wastewaters. Environ. Sci. Technol..

[bib24] Martins T.H., Souza T.S., Varesche M.B.A. (2018). The Influence of stirring speed, temperature and initial nitrogen concentration on specific anammox activity. Braz. Arch. Biol. Technol..

[bib25] Moral Pajares E., Gallego Valero L., Román Sánchez I.M. (2019). Cost of urban wastewater treatment and ecotaxes: evidence from municipalities in southern Europe. Water.

[bib26] Musa M.A., Idrus S. (2020). Effect of hydraulic retention time on the treatment of real cattle slaughterhouse wastewater and biogas production from HUASB reactor. Water.

[bib27] Nayl A.E.A., Elkhashab R.A., Malah T., Yakout S.M., El-khateeb M.A., Ali M.M.S., Ali H.M. (2017).

[bib28] Neoh C.H., Lam C.Y., Ghani S.M., Ware I., Sarip S.H.M., Ibrahim Z. (2016). Bioremediation of high-strength agricultural wastewater using Ochrobactrum sp. strain SZ1. 3. Biotech.

[bib29] Ölmez T. (2009). The optimization of Cr (VI) reduction and removal by electrocoagulation using response surface methodology. J. Hazard Mater..

[bib30] Rahman A.S. (2019). Nanotechnology in Eco-Efficient Construction.

[bib31] Rajasulochana P., Preethy V. (2019). Sewage treatment by using green algae Scenedesmus, Chlorella and their combination. Desalination Water Treat..

[bib32] Sa’at S.K.M., Zaman N.Q., Yusoff M.S. (2019). AIP Conference Proceedings.

[bib33] Salama Y., Chennaoui M., Sylla A., Mountadar M., Rihani M., Assobhei O. (2014). Review of wastewater treatment and reuse in the Morocco: aspects and perspectives. Int. J. Environ. Pollut. Res..

[bib34] Samer M. (2015). Biological and chemical wastewater treatment processes. Wastewater Treatment Eng..

[bib35] Sonawane S., Setty Y.P., Sapavatu S.N. (2015). Chem. Bioprocess Eng.: Trends Dev..

[bib36] Sikosana M.L., Sikhwivhilu K., Moutloali R., Madyira D.M. (2019). Municipal wastewater treatment technologies: a review. Procedia Manuf..

[bib37] Valdivia A., Gonzalez-Martinez S., Wilderer P.A. (2007). Biological nitrogen removal with three different SBBR. Water Sci. Technol..

[bib38] Zhao W., Yu Z., Liu J., Yu Y., Yin Y., Lin S., Chen F. (2011). Optimized extraction of polysaccharides from corn silk by pulsed electric field and response surface quadratic design. J. Sci. Food Agric..

